# Meta-Analysis and Experimental Studies Reveal Mitotic Network Activity Index (MNAI) as Breast Cancer Metastasis and Treatment Biomarker

**DOI:** 10.3390/life15121931

**Published:** 2025-12-17

**Authors:** Yimeng Cai, Chun Fung Kwok, Hang Chang, Jian-Hua Mao

**Affiliations:** 1 Biological Systems and Engineering Division, Lawrence Berkeley National Laboratory, Berkeley, CA 94720, USA; cym2020apply@163.com (Y.C.); cf.kwok02@connect.hku.hk (C.F.K.); hchang@lbl.gov (H.C.); 2 Berkeley Biomedical Data Science Center, Lawrence Berkeley National Laboratory, Berkeley, CA 94720, USA

**Keywords:** breast cancer, biomarkers, distant metastasis free survival, treatment response, cell migration and invasion

## Abstract

**Objective:** Identifying biomarkers that predict metastatic potential or guide treatment selection is critical for improving breast cancer (BC) management. Previously, we established the Mitotic Network Activity Index (MNAI) as a prognostic marker in BC. Here, we bioinformatically and experimentally evaluated MNAI as a biomarker for metastasis risk and therapeutic response. **Methods:** We used Kaplan–Meier and Cox proportional hazard regression analyses to assess the association between MNAI and distant metastasis-free survival (DMFS) across 14 published BC datasets. A total of 16 publicly available clinical trial datasets, including the I-SPY trials, were used to evaluate the predictive value of MNAI for treatment response. Additionally, wound-healing and transmembrane assays were conducted to determine the effects of PLK1, CHEK1, and BUB1 inhibition on BC cell migration and invasion. **Results:** High MNAI levels were strongly associated with shorter DMFS. Multivariate analysis further confirmed MNAI as an independent risk factor for DMFS, beyond estrogen receptor status and PAM50-based molecular subtypes. Functionally, pharmacologic disruption of the mitotic network using PLK1, CHEK1, or BUB1 inhibitors significantly reduced cell migration and invasion in MDA-MB-231 and BT-549 BC cell lines. Moreover, BC cells with high MNAI increased sensitivity to microtubule-targeting agents such as docetaxel, paclitaxel, and ixabepilone but increased resistance to tamoxifen, AKT1/2 inhibitors, and mTOR inhibitors. Consistent with these findings, analysis of 16 clinical trial cohorts revealed that patients with high MNAI achieved higher pathological complete response rates to taxane-containing and ixabepilone-based therapies. **Conclusions:** Our findings demonstrate the MNAI as a clinically actionable biomarker that can refine risk stratification and guide the selection of targeted or chemotherapy regimens, advancing precision medicine in BC management.

## 1. Introduction

It is well-known that breast cancer (BC) wreaks havoc in American women’s lives [1]. Despite significant advances in early detection and therapy, tumor metastasis remains the true driver of mortality, accounting for the vast majority of deaths [2,3,4]. Therefore, identifying the metastatic potential of a tumor is crucially important to oncologists, which considerably influences their decision-making about cancer treatment. Over the past two decades, tremendous effort has been made to characterize the cancer omics for discovering biomarkers significantly associated with prognosis of BC patients [5,6,7]. However, limited progress has been made in developing biomarkers that more accurately predict metastatic risk. Such biomarkers could enable personalized treatment strategies that minimize both overtreatment and undertreatment, while also paving the way for novel clinical interventions aimed at preventing metastasis by targeting key molecular drivers. Furthermore, clinical responses to anticancer therapies vary considerably among individuals and are often restricted to a subset of patients [8]. Many patients with BC are subjected to unnecessary aggressive therapy from which they are unlikely to benefit because of the inadequacy in reliable biomarkers for predicting outcome. So, there is an emerging need for biomarkers to predict responses to cancer therapy.

One critical biological hallmark of cancer is dysregulated cell division (mitosis). Normal mitotic progression requires the coordinated activity of structural proteins, motor molecules, and regulatory kinases and phosphatases. Perturbations in these mitotic regulators can lead to genomic instability [9] and subsequently lead to carcinogenesis [9,10,11,12]. It has been reported that many mitotic genes are overexpressed in many types of human cancer [9,10,11,12]. Additionally, many studies show that cancer cells are more susceptible to mitotic inhibition, so many mitotic inhibitors have been developed for cancer treatment [13]. However, it is important to develop molecular markers that distinguish which tumors will be most responsive to mitotic inhibitors.

We and others have previously identified a mitotic network that is conserved across both human and murine tumors [14,15,16,17]. From this, we defined a Mitotic Network Activity Index (MNAI) and showed that the tumors with higher MNAI are likely to be preferentially sensitive to mitotic protein inhibitors [14]. Moreover, MNAI has recently been reported as a prognostic biomarker in BC and other cancer types [14,16]. Furthermore, emerging evidence shows that many mitotic genes also play critical roles in cancer metastasis and response to therapy [11,18]. For example, Aurora kinase A (AURKA) promotes epithelial–mesenchymal transition, facilitating invasion and metastasis [19,20,21,22].

In this study, we hypothesized that MNAI serves both as a prognostic biomarker for metastasis and a predictive biomarker for treatment response in BC. To test this hypothesis, we conducted a meta-analysis across BC cohorts and clinical trial studies to evaluate the predictive value of MNAI for distant metastasis development and treatment response. Additionally, we examined whether pharmacological inhibition of mitotic network components attenuates invasive cellular phenotypes in vitro. This study provides the first comprehensive meta-analysis and experimental validation, which demonstrates that the MNAI functions not only as a prognostic marker of metastatic risk but also as a predictive biomarker of therapeutic response in BC. This study advances prior MNAI research by bridging genome-scale network analysis, clinical outcomes, and therapeutic response, offering a new framework for precision oncology-guided treatment stratification in BC.

## 2. Materials and Methods

### 2.1. Study Design and Cohorts

In this cohort-based meta-evaluation study, we collected data from previously published BC datasets based on the following criteria: The BC cohort with complete information of distant metastasis free survival (DMFS) and available transcriptomic profiles based on Affymetrix Human U133A or U133plus2 were included. Cohorts lacking essential metadata or with fewer than 50 samples were excluded. A total of 14 cohorts were finally chosen for this study (Table S1). These 14 cohorts comprised 2802 BC patients. Among them, 693 patients developed distant metastasis (Table S2). A total of 16 publicly available datasets from different clinical trials or studies including I-SPY trails were used to evaluate MNAI as a biomarker for predicting the pathological complete response (pCR) rate of different treatment regimens (Table S3). All these datasets were directly downloaded from the Gene Expression Omnibus (GEO) repository (https://www.ncbi.nlm.nih.gov/gds) (accessed on 30 April 2024). No additional modifications were made to the downloaded transcriptomic data during our analysis.

### 2.2. MNAI Calculation and Categorization

MNAI was calculated as described in the previous study [14], i.e., the sum of the transcriptional levels of the 54 coordinately regulated mitotic apparatus genes. The Affymetrix probe ID of each gene is listed in Table S4. If a gene had multiple probes, the transcriptional level of the gene was the average of all probe values. For DMFS, MNAI was dichotomized within each cohort at the median, with values above the median classified as “high MNAI” and those at or below classified as “low MNAI”. Subsequently, we pooled the high- and low-MNAI groups across all cohorts to make a single group of patients. For pCR, patients were divided into three groups based on MNAI, i.e., a group with high (top one-third), intermediate (middle one-third), and low (bottom one-third) MNAI.

### 2.3. Cell Lines, Cell Culture, and Drug Treatment

The human BC cell lines MDA-MB-231 and BT-549 were purchased from the ATCC (American Type Culture Collection, Manassas, VA, USA). Cell culture was performed following the manufacturer’s protocol. We selected MDA-MB-231 and BT549 because TNBC represents the subtype with the highest MNAI and poorest prognosis in our cohort analyses. These lines are widely used and well-characterized models for studying metastatic behavior. All cells were cultured at 37 °C in the incubator supplied with 5% CO_2_. PLK1 (Volasertib), BUB1 (BAY-1816032), and CHEK1 (Rabusertib) inhibitors were purchased from the Selleckchem (Houston, TX, USA), were dissolved in DMSO to make a 10 mM stock solution, and were diluted in cell culture medium at different final concentrations to treat cells.

### 2.4. Wound-Healing Assay

The effects of the mitotic gene inhibitor on cell migratory ability were measured by scratch and transmembrane assays. For the scratch assay, cells were cultured in 12-well plates. When about 100% of the surface was occupied, a straight scratch was made to remove cells by a 1 mL pipette tip. After washing with medium twice, the separated cells were re-cultured in the medium with 1% FBS and different concentrations of mitotic gene inhibitor. Images were acquired at 0 and 24 h using a Nikon Eclipse TS100 inverted microscope (Marshall Scientific, Hampton, NH, USA), and wound closure was quantified as the percentage of the scratched area repopulated by cells.

### 2.5. Transwell Assay

The effects of mitotic gene inhibitors on cell migration and invasion were assessed using the Transwell assay. For migration assay, a membrane inset for 24 well was used to establish two compartments in a well, and 10^4^ cells were placed in the upper chamber, while for the invasion assay, the QCM ECMatrix cell invasion kit (MilliporeSigma, Rockville, MD, USA) was used, and 5 × 10^4^ cells were added to the upper compartment. An amount of 700 µL media with 10% FBS was added into the lower compartment. The same concentration (100 nM) of mitotic gene inhibitor was added in both the upper and lower compartments. Cells were cultured at 37 °C with a 5% CO_2_ humidified atmosphere for 24 h. The remaining cells on the upper surface were gently removed using a cotton swab. Then, the membranes were cleaned with PBS; cells were fixed with 100% methanol for 10 min and stained with 0.5% crystal violet for 20 min.

For the above cell migration and invasion assay, we performed triplicate measurements at each dose per experiment. All experiments were repeated three times.

### 2.6. Statistical Analysis

Association of MNAI with DMFS was assessed by Kaplan–Meier plot and Cox proportional hazard regression analysis in each cohort and pooled data. Our meta-analysis employed an aggregated-data approach, using summary statistics from each individual study. For each cohort, patients were first stratified into high-MNAI (above the median) and low-MNAI (at or below the median) groups, and DMFS outcomes were analyzed separately within each dataset. Subsequently, we pooled the high- and low-MNAI groups across all cohorts to perform an integrated meta-analysis, thereby assessing the overall prognostic impact of MNAI across independent studies. Heterogeneity measures to assess the dissimilarity between data sets were conducted in R (version 4.3.1). The independent predictive value of MNAI for DMFS was examined by multivariant Cox proportional hazard regression analysis. Difference in DMFS between high- and low-MNAI groups was assessed by hazard ratio and log rank test. A nomogram was created to investigate the clinical significance of MNAI together with PAM50 and estrogen receptor (ER) status, and c-index and calibration curves were used to evaluate the performance of a predictive model by R scripts (version 4.3.1). The association of MNAI with pCR was assessed by Chi-Square test. Difference in cell migration and invasive capabilities between treatment groups were assessed by *t*-test. The association between drug IC50 and MNAI in BC cell lines was examined by Spearman correlation. All statistical tests were two-sided, with statistical significance defined as *p* < 0.05. All statistical analyses were conducted, and the Figure panels were generated using SPSS (IBM SPSS Statistics 24).

## 3. Results

### 3.1. MNAI Is a Significantly and Independently Predictive Biomarker for DMFS of BC Patients

**Figure 1:**
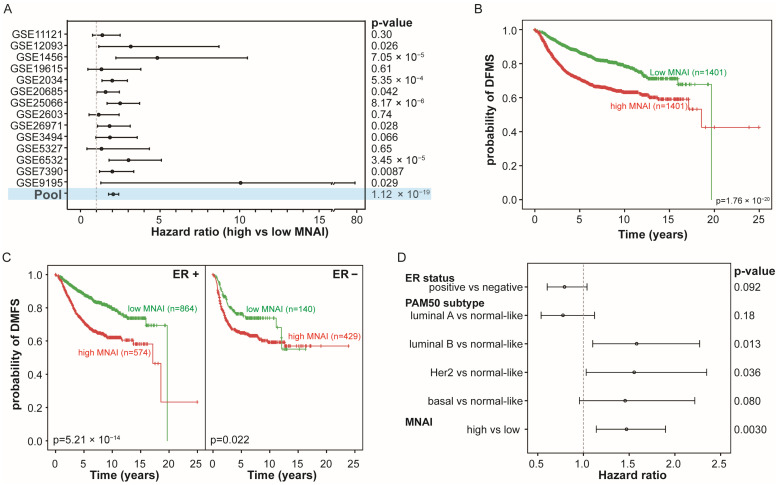
Association of MNAI with DMFS in BC patients. (**A**) Forest plot for hazard ratios (HRs) by showing each cohort’s HR as a central point and its 95% confidence interval as a horizontal line. (**B**) Kaplan–Meier curves of DMFS in the pooled dataset by combining 14 cohorts. (**C**) Kaplan–Meier curves of DMFS stratified by ER status. (**D**) Forest plot for HRs by showing each clinical factor’s HR as a central point and its 95% confidence interval as a horizontal line. The *p* values were obtained by log rank test.

**Figure 2:**
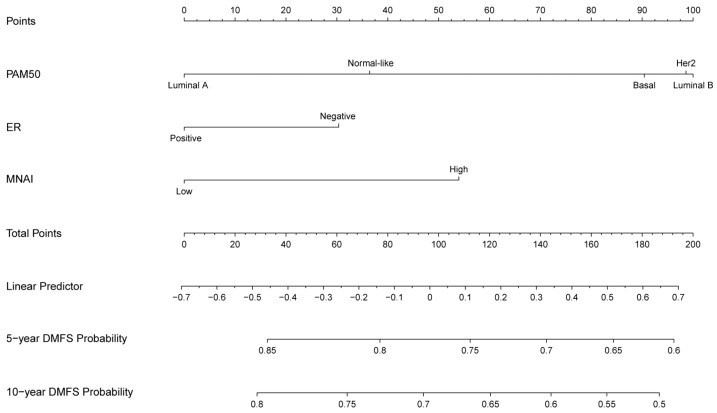
Prognostic nomogram using the MNAI, ER, and PAM50 to predict 5- and 10-year DMFS in BC patients.

We examined the association of MNAI with DMFS across a large cohort of 2802 BC patients from 14 publicly available datasets (Table S1). Within each dataset, patients were stratified into high- and low-MNAI groups based on the cohort-specific median of MNAI (for details, see Section 2). Across nearly all datasets, patients with high MNAI consistently exhibited shorter DMFS compared with those with low MNAI, reaching statistical significance in 10 out of 14 cohorts (*p* < 0.05, Figure 1A and Figure S1). When all datasets were pooled into a single meta-cohort, high MNAI remained strongly associated with increased metastatic risk (HR = 2.06, 95% CI: 1.76–2.40, *p* = 1.12 × 10^−19^, Figure 1A,B). Heterogeneity analysis revealed that the observed variation across studies is much smaller than would be expected by chance (I^2^ = 0 and *p* = 0.9999). Moreover, high MNAI was significantly associated with shorter DMFS in both ER-positive and ER-negative patients (*p* = 5.21 × 10^−14^ and 0.022, respectively, Figure 1C). Importantly, multivariate Cox proportional hazard regression analysis revealed that MNAI is an independent risk factor for DMFS, retaining significance after adjusting for ER status and PAM50 molecular subtype (Figure 1D, Table S5). Finally, nomogram-based survival modeling revealed that integrating MNAI with ER status and PAM50 classification substantially improved the accuracy of 5-year and 10-year DMFS prediction in BC patients (Figure 2). This enhanced predictive performance was further supported by both c-index analysis (Figure S2) and calibration curves (Figure S3).

Collectively, these findings indicate that elevated MNAI is a robust, independent predictor of distant metastasis in BC and may serve as a clinically informative biomarker for risk stratification.

### 3.2. Mitotic Gene Inhibitors Suppress BC Cell Migration and Invasion In Vitro

To further assess the functional relevance of MNAI as a metastatic biomarker, we experimentally examined whether perturbing key components of the mitotic network influences cell motility and invasiveness. Specifically, we targeted three representative mitotic regulators, i.e., BUB1, CHEK1, and PLK1, using selective small-molecule inhibitors and evaluated their effects on BC cell migration and invasion through multiple complementary assays.

**Figure 3:**
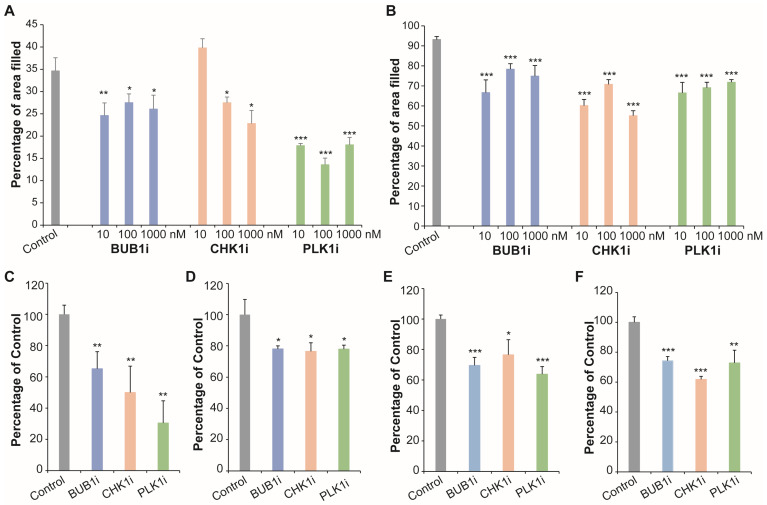
Effect of mitotic gene inhibitors on BC cell migration and invasion. (**A**,**B**) Cell migration was determined by wound-healing assay in MDA-MB-231 (**A**) and BT-549 (**B**) cell lines. (**C**,**D**) Cell migration was determined by Transwell assay in MDA-MB-231 (**C**) and BT-549 (**D**) cell lines. (**E**,**F**) Cell invasiveness was determined by Matrigel-coated Transwell assay in MDA-MB-231 (**E**) and BT-549 (**F**) cell lines. There were three replicates per treatment. The *p* values were obtained using t-test by comparing treatment group to control group. * *p* < 0.05; ** *p* < 0.01; and *** *p* < 0.001.

In the wound-healing assay, treatment with inhibitors of BUB1, CHEK1, or PLK1 significantly delayed wound closure in both MDA-MB-231 and BT-549 triple-negative BC cell lines, indicating impaired migratory capacity (Figure 3A,B, Table S6). The inhibitory effect was not statistically significantly different across all tested compounds and concentrations, showing no clear dose–response relationship (Figure 3A,B), which justified the use of 100 nM as a standardized concentration for subsequent experiments.

Using Transwell migration and Matrigel invasion assays, we further demonstrated that inhibition of each mitotic regulator markedly reduced both cell migration (Figure 3C,D, Table S6) and invasive potential (Figure 3E,F, Table S6). These consistent findings across independent assays suggest that mitotic network activity contributes not only to proliferative signaling but also to the cellular mechanisms underlying metastatic dissemination.

Collectively, these results support the concept that MNAI reflects a biologically active mitotic signaling axis that promotes tumor cell motility and invasion, reinforcing its potential utility as a predictive biomarker of metastatic risk in BC. Moreover, the sensitivity of invasive phenotypes to mitotic inhibition highlights possible therapeutic avenues for targeting mitosis-associated pathways to suppress metastasis in high-MNAI tumors.

### 3.3. Correlation Between MNAI and pCR Rate of BC Treatments in Multiple Cohorts

**Figure 4:**
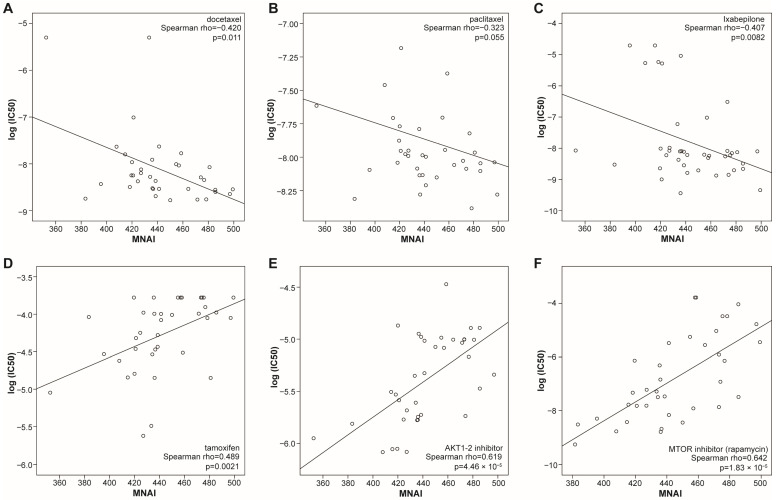
Association of MNAI with IC50 of different treatments in breast cancer cell lines. (**A**) Docetaxel. (**B**) Paclitaxel. (**C**) Ixabepilone. (**D**) Tamoxifen. (**E**) AKT1-2 inhibitor. (**F**) mTOR inhibitor (rapamycin). Spearman correlation was used to assess the relationship between IC50 and MNAI.

Previous work demonstrated that BC cells with high MNAI exhibit increased susceptibility to inhibitors targeting key mitotic regulators such as PLK1, CENPE, and AURKB/C [14]. Building upon these observations, we systematically explored the broader relationship between MNAI and drug sensitivity across a panel of well-characterized BC cell lines [21]. Using pharmacogenomic profiling data, we found that cell lines with elevated MNAI were significantly more sensitive to microtubule-targeting chemotherapeutic agents, including docetaxel, paclitaxel, and ixabepilone (Figure 4A–C, Table S7). These findings suggest that tumors with high MNAI may harbor intrinsic vulnerabilities to agents that disrupt spindle dynamics and cell-cycle progression. Conversely, high-MNAI BC cell lines demonstrated reduced sensitivity (or relative resistance) to endocrine therapy (tamoxifen) and targeted agents acting on the AKT–mTOR signaling axis (AKT1/2 and mTOR inhibitors) (Figure 4D–F, Table S7). This pattern implies that tumors driven by elevated mitotic signaling may be less dependent on hormone receptor-mediated or PI3K/AKT–mTOR pathways for growth and survival, highlighting a potential mechanistic shift toward mitotic dominance in their proliferative programs.

To evaluate the clinical relevance of these in vitro findings, we extended our analysis to 16 publicly available BC patient cohorts, including several large-scale datasets such as the I-SPY clinical trials. Consistently, patients with high-MNAI tumors exhibited a significantly higher pathologic complete response (pCR) rate to taxane-containing chemotherapy regimens and ixabepilone treatment (Table 1). Importantly, after adjusting available clinical factors by multivariate logistic regression analysis, we found that MNAI is still significantly associated with pCR in majority of clinical datasets, indicating that MNAI is an independent predictor of pCR (Table S8).

Taken together, and integrating these clinical observations with our experimental data, we propose that MNAI serves as a promising predictive biomarker for therapeutic responses, identifying tumors that are preferentially sensitive to microtubule-stabilizing agents and resistant to hormonal or PI3K/AKT-mTOR-targeted therapies. This functional stratification underscores the potential utility of MNAI in guiding personalized therapy selection and optimizing treatment outcomes in BC.

## 4. Discussion

In this study, we provide robust evidence that the MNAI is a clinically meaningful biomarker of BC progression and treatment response. By integrating data from 14 independent cohorts encompassing 2802 patients, we showed that high MNAI is strongly and statistically significantly associated with worse DMFS, independent of established clinicopathologic variables such as ER status and PAM50 molecular subtype. These findings suggest that MNAI captures a fundamental proliferative and metastatic program beyond conventional biomarkers, indicating its potential value in clinical decision-making.

The prognostic impact of high MNAI observed in our pooled analysis highlights the central role of mitotic dysregulation in BC progression. Our findings are consistent with prior studies linking aberrant mitotic checkpoint activation, chromosomal instability, and aneuploidy to tumor aggressiveness and poor outcomes [10,11,16,18,19,20]. Importantly, the independent prognostic power of MNAI relative to ER and PAM50 subtype classification indicates that MNAI provides additive information that may refine existing risk stratification models. Clinically, incorporation of MNAI into prognostic nomograms may help identify patients at increased risk of distant recurrence who could benefit from intensified systemic therapy or closer surveillance.

Our functional assays further support the biological relevance of MNAI by showing that pharmacologic inhibition of key mitotic regulators (PLK1, CHEK1, BUB1) significantly reduced cell migration and invasion. These results suggest that MNAI not only reflects proliferative potential but also underlies metastatic behavior through mitotic checkpoint dysregulation [18,19,20,23]. This mechanistic link between mitotic network activation and invasion provides a rationale for therapeutic targeting of mitotic regulators in high-MNAI tumors.

The analysis across 16 clinical trial datasets revealed a striking pattern of treatment response associated with MNAI. The observed association between high MNAI and increased taxane sensitivity is biologically plausible given the central role of mitotic network integrity in determining cellular vulnerability to microtubule-targeting agents [13,24,25,26]. Tumors with elevated MNAI exhibit heightened reliance on the spindle assembly checkpoint (SAC) to maintain chromosomal stability despite persistent mitotic stress [27]. Taxanes exacerbate this instability by disrupting microtubule dynamics, prolonging mitotic arrest, and overwhelming an already strained checkpoint apparatus [26,28]. In cancers with pre-existing mitotic dysregulation, such as defects in spindle organization, kinetochore–microtubule attachment, or checkpoint signaling, taxane-induced stress more readily triggers apoptosis rather than mitotic slippage, thereby enhancing therapeutic response [28]. This mechanistic framework aligns with prior reports linking SAC dependency, chromosomal instability, and aberrant mitotic spindle architecture to taxane vulnerability, further supporting MNAI as a biologically grounded predictor of taxane efficacy. In vitro studies showed that high MNAI makes cells resistant to tamoxifen, AKT inhibition, or mTOR inhibition. These findings are consistent with the mechanism of action of mitotic-targeting agents, which preferentially affect rapidly proliferating tumor cells with high mitotic checkpoint dependency [29,30,31,32]. In contrast, endocrine and PI3K-AKT-mTOR pathway inhibitors may be less effective in this subgroup due to intrinsic proliferative dominance and reduced reliance on hormonal or growth factor signaling [33,34,35,36].

From a translational perspective, MNAI could serve as a predictive biomarker to guide treatment selection. Specifically, patients with high-MNAI tumors may derive substantial benefit from mitotic-targeting agents such as taxanes or microtubule-stabilizing drugs, while those with low-MNAI tumors may respond better to endocrine or PI3K–AKT-mTOR-targeted therapies. Prospective validation of MNAI in biomarker-stratified clinical trials will be necessary to confirm its utility in optimizing treatment selection.

In contrast to established gene signatures such as Oncotype DX and MammaPrint, which primarily quantify proliferation, hormone signaling, and broad transcriptional programs, MNAI captures a distinct and mechanistically focused dimension of tumor biology: the activity of mitotic machinery and its associated genomic instability. Whereas many existing signatures rely on diverse, pathway-agnostic gene sets, MNAI is derived from a well-defined network of mitotic regulators, enabling clearer biological interpretability and potentially greater relevance for predicting response to mitosis-targeting therapies such as taxanes. From a feasibility standpoint, MNAI offers practical advantages for clinical deployment. The index can be computed directly from standard RNA-seq or microarray data without requiring proprietary algorithms, reducing cost and facilitating implementation across laboratories. Because it depends on a concise and biologically coherent gene network, MNAI is amenable to assay standardization, including RT-qPCR panels or targeted sequencing platforms. However, successful translation will require validation across diverse clinical settings, assessment of assay reproducibility, and calibration to ensure compatibility with existing risk-stratification tools. Together, these features position MNAI as a cost-effective and mechanistically grounded complement to current genomic assays.

Several limitations should be acknowledged. First, although our use of multiple independent cohorts and standardized analytical pipelines reduces the likelihood of overfitting, the integration of heterogeneous transcriptomic datasets inherently carries this risk. Differences in cohort composition, microarray platforms, and preprocessing methods may also introduce variability that could influence model performance. Second, the publicly available datasets included in our pooled analyses may be subject to publication bias, as studies with more complete annotation or stronger biological signals are more likely to be deposited and cited. This could lead to an overrepresentation of BC subtypes or study designs. While our cross-cohort validation helps mitigate these concerns, future work incorporating prospective, population-based datasets will be essential for confirming the generalizability of our findings. Third, while we observed consistent patterns across multiple datasets, multi-institutional prospective clinical validation of MNAI as both a prognostic and predictive biomarker is required before clinical implementation. Fourth, our experimental assays focused on a limited number of mitotic regulators; additional studies are warranted to define the broader mechanistic landscape of MNAI and its role in metastatic progression. Finally, although MNAI was developed and validated in BC, it remains unclear whether this index has predictive value in other tumor types.

## 5. Conclusions

This study establishes MNAI as a powerful biomarker for both metastasis risk and therapeutic response in BC. By reflecting fundamental mitotic dysregulation, MNAI provides independent prognostic information and predicts sensitivity to mitotic-targeting chemotherapies. Our findings highlight the translational potential of MNAI to improve risk stratification, guide therapeutic selection, and ultimately advance precision management of BC patients.

## Figures and Tables

**Table 1:** Association of MNAI with the response to different treatments in breast cancer patients.

GEO Dataset	Study	Type of Therapy	Number of Patients	Number of pCR	MNAI			*p* Value
					Low	Intermediate	High	
GSE4779	A phase III clinical trial (EORTC 10994)	FEC *	102	39	34 (29.41%) ^†^	34 (44.12%)	34 (41.18%)	0.42
GSE16446	The neoadjuvant TOP trial	epirubicin	114	16	39 (12.82%)	38 (7.89%)	37 (21.62%)	0.22
GSE20194	MDACC 2003-0321 trail	TFAC *	214	48	68 (4.41%)	70 (25.71%)	76 (35.53%)	**3.34 × 10^−5^**
GSE20271	MAQC2	FAC/FEC *	87	7	28 (0%)	29 (13.79%)	30 (10%)	0.14
		TFAC/TFEC *	91	19	31 (9.68%)	31 (22.58%)	29 (31.03%)	0.12
GSE22226	I-SPY1	AC+T *	107	27	35 (11.43%)	37 (24.32%)	35 (40%)	**0.022**
GSE23988	USO 02103	FAC/TX *	61	20	20 (5%)	21 (28.57%)	20 (65%)	**2.49 × 10^−4^**
GSE25066	Multicenter prospective study	taxane+anthracycline	488	99	160 (5.63%)	164 (18.9%)	164 (35.98%)	**8.30 × 10^−11^**
GSE32646	Retrospective study	TFEC	115	27	38 (2.63%)	39 (30.77%)	38 (36.84%)	**8.57 × 10^−4^**
GSE41998	A randomized, open-label, multicenter, phase II trial (NCT00455533)	Ixabepilone	138	35	54 (16.67%)	43 (34.88%)	41 (26.83%)	0.12
		Paclitaxel	127	34	37 (17.14%)	43 (29.79%)	41 (35.56%)	0.18
GSE42822	USO 02103	FEC/TX	66	25	22 (18.18%)	23 (34.78%)	21 (61.9%)	**0.012**
GSE50948	The NeoAdjuvant Herceptin [NOAH] trial	AT+CMF *	93	22	29 (10.34%)	35 (28.57%)	29 (31.03%)	0.12
		AT+CMF+Trastuzumab	63	31	23 (34.78%)	17 (61.54%)	23 (65.22%)	0.12
		Pooled	156	53	52 (21.15%)	52 (34.62%)	52 (46.15%)	**0.027**
GSE66399	CHER-LOB	TFEC+trastuzumab	23	6	6 (0%)	9 (11.11%)	8 (62.5%)	**0.013**
		TFEC+lapatinib	31	8	13 (7.69%)	8 (0%)	10 (70%)	**4.98 × 10^−4^**
		TFEC+trastuzumab+lapatinib	34	13	10 (20%)	13 (7.69%)	11 (90.91%)	**5.93 × 10^−5^**
		Pooled	88	27	29 (10.34%)	30 (6.67%)	29 (75.86%)	**9.22 × 10^−10^**
GSE140494	EudraCT: 2008-006381-29	DFEC *	91	23	30 (16.67%)	31 (16.13%)	30 (43.33%)	**0.021**
GSE163882	Multicenter retrospective study	taxane-based chemotherapy	222	80	74 (22.97%)	74 (32.43%)	74 (52.7%)	**6.07 × 10^−4^**
GSE173839	I-SPY2	paclitaxel+durvalumab+olaparib	71	29	24 (12.5%)	26 (42.31%)	21 (71.43%)	**3.14 × 10^−4^**
GSE194040	I-SPY2	paclitaxel	179	31	55 (7.27%)	61 (18.03%)	63 (25.4%)	**0.034**
		Paclitaxel+ABT888+Carboplatin	71	27	13 (0%)	25 (40%)	33 (51.52%)	**0.0051**
		Paclitaxel+AMG386	115	33	33 (21.21%)	37 (24.32%)	45 (37.78%)	0.22
		Paclitaxel+AMG386+Trastuzumab	19	6	11 (27.27%)	6 (50%)	2 (0%)	0.38
		Paclitaxel+Ganetespib	93	26	22 (8.7%)	23 (18.18%)	48 (41.67%)	**0.0076**
		Paclitaxel+Ganitumab	106	24	29 (3.45%)	40 (25%)	37 (35.14%)	**0.0085**
		Paclitaxel+MK-2206	60	18	18 (27.78%)	20 (30%)	22 (31.82%)	0.96
		Paclitaxel+MK-2206+Trastuzumab	34	17	12 (50%)	17 (52.94%)	5 (40%)	0.88
		Paclitaxel+Neratinib	114	42	42 (26.19%)	34 (47.06%)	38 (39.47%)	0.16
		Paclitaxel+Pembrolizumab	69	31	28 (25%)	20 (45%)	21 (71.43%)	**0.0054**
		Paclitaxel+Pertuzumab+Trastuzumab	44	26	25 (48%)	14 (71.43%)	5 (80%)	0.22
		Paclitaxel+Trastuzumab	31	8	18 (22.22%)	9 (44.44%)	4 (0%)	0.21
		T-DM1+Pertuzumab	52	30	22 (45.45%)	24 (70.83%)	6 (50%)	0.20

* FEC: 5-fluorouracil+epirubicin+cyclophosphamide; TFEC: paclitaxel+5-fluorouracil+epirubicin+cyclophosphamide; FAC: 5-fluorouracil+doxorubicin+cyclophosphamide; TFAC: paclitaxel+5-fluorouracil+doxorubicin+cyclophosphamide; AC+T: Anthracycline+Cyclophosphamide+Docetaxel; TX: docetaxel+capecitabine; AT+CMF: doxorubicin/paclitaxel+cyclophosphamide/methotrexate/fluorouracil; DFEC: docetaxel+5-fluorouracil+epirubicin+cyclophosphamide. ^†^ number of patients (% of pCR). The *p* values were obtained from the Chi-Square test.

## Data Availability

All data used in this study were downloaded from public databases, which are provided in Supplementary Materials (Tables S2 and S3).

## Supplementary Materials

The following supporting information can be downloaded at: https://www.mdpi.com/article/10.3390/life15121931/s1, Figure S1: Association of MNAI with DMFS in each dataset; Figure S2: Evaluation of nomogram-based DMFS model’s performance by c-index; Figure S3: Evaluation of nomogram-based DMFS model’s performance by calibration curve; Table S1: List of datasets used for assessing relationship between MNAI and distant metastasis-free survival; Table S2: Patients used for association of MNAI with DMFS (to generate Figure 1); Table S3: All datasets used for examining the association of MNAI with pCR; Table S4: The Affymetrix probe ID of each MNAI gene; Table S5: Association of MNAI with DMFS adjusted by clinical factors in human breast cancer; Table S6: Statistical summary results for cell migration and invasion; Table S7: Correlation between IC50 and MNAI in human breast cancer cell lines; Table S8: Association of MNAI with pCR adjusted by clinical factors in different clinical datasets.

## Abbreviations

AURKA	aurora kinase A
BC	breast cancer
CI	confidence interval
DMFS	distant metastasis free survival
HR	hazard ratio
IC50	half-maximal inhibitory concentration
MNAI	Mitotic Network Activity Index
pCR	pathological complete response
RT-qPCR	reverse transcription quantitative polymerase chain reaction
SAC	spindle assembly checkpoint
