# Supplementary material for: Meta-Analysis and Experimental Studies Reveal Mitotic Network Activity Index (MNAI) as Breast Cancer Metastasis and Treatment Biomarker
Source: Life (Basel). 2025 Dec 17;15(12):1931. doi: 10.3390/life15121931 (PMC12734722; doi:10.3390/life15121931)
Supplement: Supplementary file 1 [file life-15-01931-s001.zip › MNAI_BC_SupplementalMaterials.pdf]

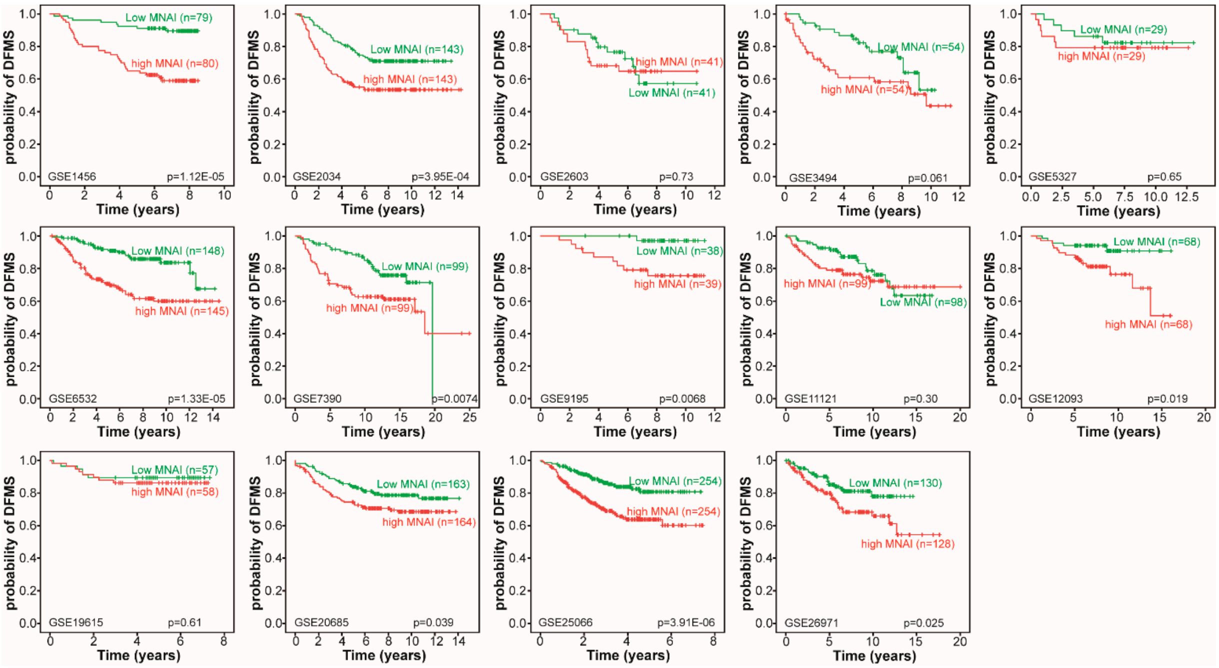

Figure S1 Association of MNAI with DMFS in each dataset. Kaplan-Meier curves of DMFS in each dataset, and p values were obtained from log rank tests.

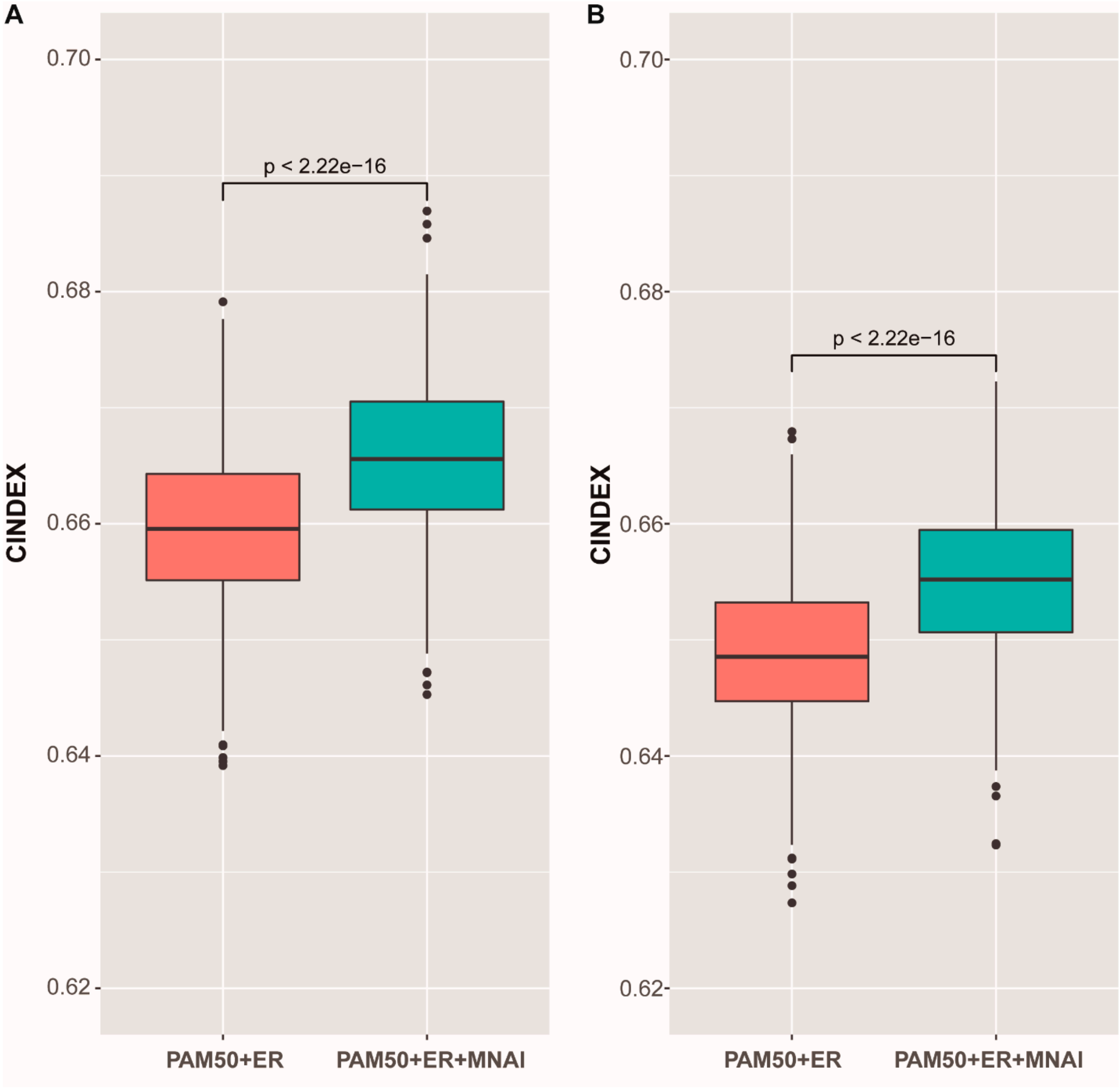

Figure S2 Evaluation of nomogram-based DMFS model's performance by c-index. (**A&B**) 5-year (**A**) and 10-year (**B**) c-index specifically evaluates the nomogram-based DMFS model's performance. The p values were obtained from Mann-Whitney tests.

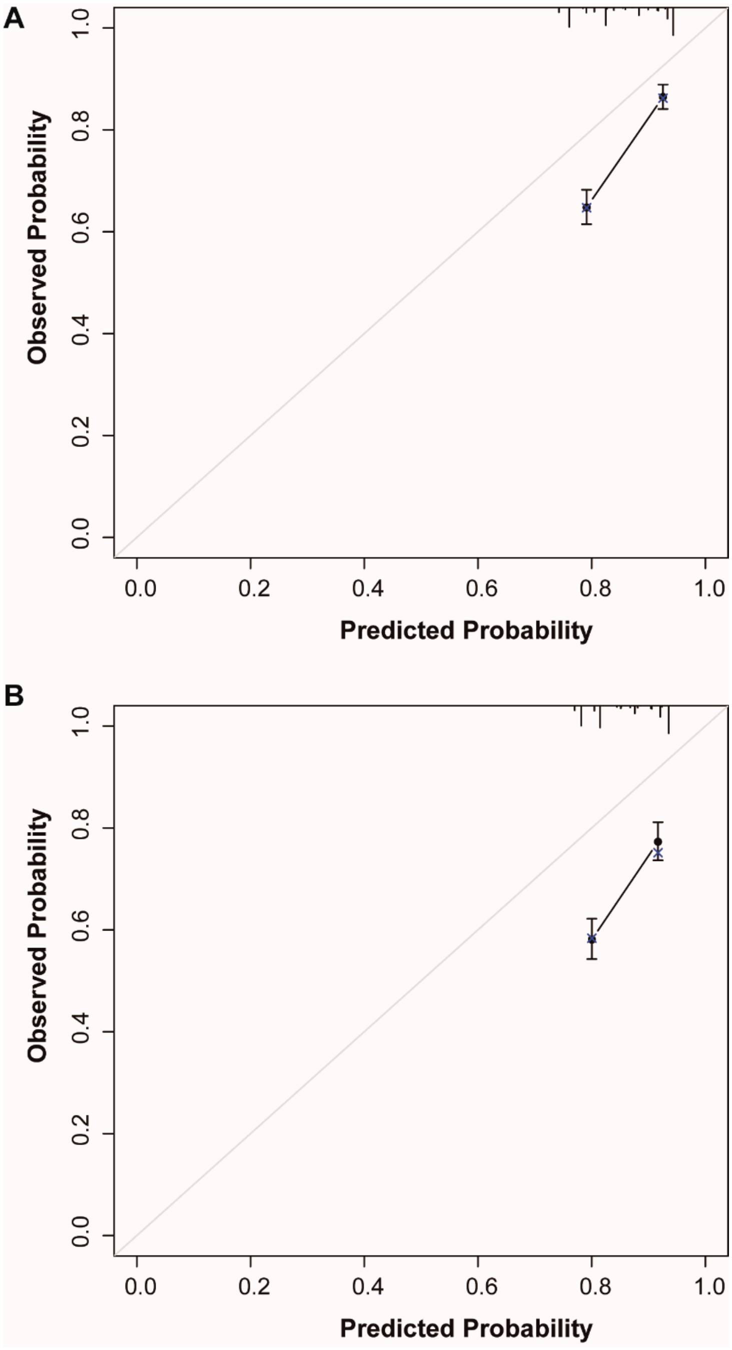

Figure S3 Evaluation of nomogram-based DMFS model's performance by calibration curve. (**A&B**) 5-year (**A**) and 10-year (**B**) calibration curve specifically evaluates the nomogram-based DMFS model's performance.

Table S1 List of datasets used for assessing relationship between MNAI and distant metastasis-free survival.

GEO dataset	Microarray type	No. patients	No. Distant metastasis event
GSE1456	U133A	159	40
GSE2034	U133A	286	107
GSE2603	U133A	82	27
GSE3494	U133A	108	38
GSE5327	U133A	58	11
GSE6532	U133A	293	68
GSE7390	U133A	198	62
GSE11121	U133A	197	44
GSE12093	U133A	136	20
GSE25066	U133A	508	111
GSE26971	U133A	258	58
GSE9195	U133plus2	77	10
GSE19615	U133plus2	115	14
GSE20685	U133plus2	327	83
**Total**		**2802**	**693**

Table S2 Patients used for association of MNAI with DMFS.

(referred to the separate excel spreadsheet: TableS2.xlsx)

Table S3 All datasets used for examining the association of MNAI with pCR.

(referred to the separate excel spreadsheet: TableS3.xlsx)

Table S4 The Affymetrix probe ID of each MNAI gene.

Gene Name	Affymetrix ID
AURKA	208079_s_at
AURKB	209464_at
BUB1	209642_at
CENPE	205046_at
FOXM1	202580_x_at
MELK	204825_at
PBK	219148_at
PLK1	202240_at
TTK	204822_at
TYMS	202589_at
ASPM	219918_s_at
BUB1B	203755_at
CCNA2	203418_at
CCNB1	214710_s_at
CCNB2	202705_at
CDC20	202870_s_at
CDCA3	221436_s_at
CDCA8	221520_s_at
CENPA	204962_s_at
CENPN	219555_s_at
CEP55	218542_at
DDX39	201584_s_at
DEPDC1	220295_x_at
DLG7	203764_at
EXO1	204603_at
FAM64A	221591_s_at
HJURP	218726_at
KIF14	206364_at
KIF18B	222039_at
KIF20A	218755_at
KIF23	204709_s_at
KIF4A	218355_at
LMNB2	216952_s_at
MAD2L1	203362_s_at
MCM10	220651_s_at
MKI67	212021_s_at
NCAPD2	201774_s_at
NCAPG2	219588_s_at
NCAPH	212949_at
NDC80	204162_at

PRC1	218009_s_at
PTTG1	203554_x_at
RFC3	204127_at
RRM2	209773_s_at
SMC4	201663_s_at
STIL	205339_at
TEX10	218104_at
TPX2	210052_s_at
UBE2S	202779_s_at
CHEK1	205393_s_at
CHEK1	205394_at
GTSE1	204317_at
GTSE1	204318_s_at
GTSE1	215942_s_at
KIF2C	209408_at
KIF2C	211519_s_at
NCAPG	218662_s_at
NCAPG	218663_at
EXOSC9	205061_s_at
EXOSC9	213226_at

Table S5 Association of MNAI with DMFS adjusted by clinical factors in human breast cancer.

Factor	Coefficient	Hazard Ratio (HR)	95% CI for HR		p value
			Lower	Upper	
**MNAI** high vs low	0.386	1.471	1.140	1.898	0.003
**PAM50** luminal A vs normal like	-0.259	0.776	0.537	1.121	0.177
**PAM50** luminal B vs normal like	0.458	1.582	1.101	2.271	0.013
**PAM50** Her2 vs normal like	0.441	1.555	1.030	2.348	0.036
**PAM50** basal vs normal like	0.376	1.456	0.956	2.218	0.080
**ER** positive vs negative	-0.233	0.793	0.604	1.039	0.092

Table S6 Statistical summary results for cell migration and invasion.

Experiment	Drug	Dose	MDA-MB-231			BT-549		
			% of area filled		p value	% of area filled		p value
			Average	Standard deviation		Average	Standard deviation	
Wound healing assay	Control	0 nM	34.63	2.97		93.17	1.52	
	iBUB1A	10 nM	24.62	2.82	0.0076	66.64	6.36	1.79E-05
		100 nM	27.50	1.97	0.041	78.36	2.74	1.80E-05
		1000 nM	26.07	3.12	0.032	74.91	5.27	2.32E-04
	iCHK1	10 nM	39.79	2.08	0.13	60.15	3.11	1.63E-10
		100 nM	27.49	1.28	0.028	70.78	2.36	1.34E-08
		1000 nM	22.82	2.94	0.018	55.09	2.51	1.43E-12
	iPLK1	10 nM	17.85	0.49	5.14E-04	66.48	5.28	2.09E-06
		100 nM	13.57	1.50	2.29E-07	69.10	2.73	1.21E-08
		1000 nM	18.03	1.67	1.33E-05	71.84	1.28	1.37E-09
Transwell migration assay	Control	0 nM	100.00	5.92		100.00	9.74	
	iBUB1	100 nM	65.24	10.81	0.0081	78.00	1.97	0.019
	iCHK1	100 nM	49.97	16.93	0.0085	76.45	5.50	0.022
	iPLK1	100 nM	30.41	14.25	0.0015	77.87	2.56	0.019
Transwell invasion assay	Control	0 nM	100.00	2.63		100.00	3.68	
	iBUB1	100 nM	69.61	5.15	8.10E-04	74.22	2.85	6.60E-04
	iCHK1	100 nM	76.46	9.91	0.016	61.84	2.07	9.69E-05
	iPLK1	100 nM	63.82	5.00	3.80E-04	72.90	8.37	0.0068

Table S7 Correlation between IC50 and MNAI in human breast cancer cell lines.

Drug	Correlation coefficient	p value
17-AAG	0.369	0.023
5-FU	-0.002	0.989
5-FdUR	0.112	0.541
AC-026-123	0.435	0.034
AC-24A	-0.157	0.454
AG1(Purvalanol)	-0.109	0.499
AG1024	0.200	0.223
AG1478	-0.135	0.433
AG2(OB-360)	-0.280	0.080
AKT1-2 inhibitor	0.619	4.461E-05
API-2(Tricir)	0.255	0.108
AS-252424	0.151	0.400
AZD6244	-0.266	0.163
BEZ235	-0.284	0.128
BI 2536	-0.250	0.147
BIBW2992	0.015	0.942
Baicalein	-0.443	0.004

Bortezomib	0.033	0.833
CPT-11	0.090	0.600
CT8	-0.088	0.669
Carboplatin	-0.070	0.670
Cetuximab	-0.297	0.093
Cisplatin	-0.181	0.263
Dasatinib	-0.141	0.411
Diaryl Urea	0.331	0.098
Docetaxel	-0.42	0.011
Doxorubicin	0.108	0.530
ERKi II (FR180304)	0.037	0.851
Epirubicin	-0.031	0.854
Erlotinib	-0.363	0.020
Etoposide	-0.34	0.032
Everolimus	0.115	0.583
FTase inhibitor I	-0.039	0.879
Fascaplysin	0.492	0.001
Fluvastatin	-0.318	0.062
Geldanamycin	-0.023	0.893
Gemcitabine	0.018	0.916
Herceptin	-0.022	0.937
Ibandronate sodium salt	0.023	0.900
Imatinib	0.301	0.055
Iressa	0.224	0.170
Ixabepilone	-0.407	0.008
Lestaurtinib(CEP-701)	-0.045	0.794
Lovastatin	-0.227	0.211
MG-132	-0.297	0.079
Methotrexate	0.072	0.676
Nilotinib	-0.192	0.445
Nutlin 3a	0.086	0.589
Nutlin 3b	0.105	0.508
Olomoucine II	0.071	0.679
Oxaliplatin	-0.191	0.245
Oxamflatin	0.191	0.258
Paclitaxel	-0.323	0.055
Pemetrexed	-0.114	0.522
Purvalanol A	0.167	0.323
Rapamycin	0.642	1.829E-05
Simvastatin	-0.393	0.020
Sorafenib	0.226	0.161
Sunitinib Malate	-0.210	0.188
Tamoxifen	0.489	0.002
Temsirolimus (Torisel)	0.319	0.055
VX-680	-0.283	0.099
Valproic acid	0.318	0.071
Vinorelbine	-0.084	0.605

Table S8 Association of MNAI with pCR adjusted by clinical factors in different clinical datasets.

Study	Factor	Odds Ratio (OR)	95% CI for OR		p value
			Lower	Upper	
GSE4779	ER positive vs negative	0.818	0.263	2.546	0.729
	PR positive vs negative	0.739	0.202	2.713	0.649
	LN positive vs negative	0.925	0.389	2.203	0.861
	**MNAI** Intermediate vs low	2.164	0.748	6.262	0.155
	**MNAI** High vs low	1.617	0.552	4.736	0.381
GSE16446	ERBB2 positive vs negative	1.599	0.485	5.267	0.440
	age ≤ 50 vs >50	1.792	0.601	5.344	0.296
	**MNAI** Intermediate vs low	0.643	0.140	2.947	0.569
	**MNAI** High vs low	2.058	0.593	7.134	0.255
GSE20194	age	0.984	0.951	1.017	0.341
	ER negative vs positive	5.355	2.163	13.262	**0.00029**
	PR negative vs positive	1.473	0.566	3.835	0.427
	HER2 negative vs positive	0.408	0.194	0.860	**0.018**
	**MNAI** Intermediate vs low	2.360	0.697	7.990	0.168
	**MNAI** High vs low	4.188	1.293	13.566	**0.017**
GSE20271	age	1.003	0.960	1.048	0.902
	ER positive vs negative	0.742	0.208	2.653	0.646
	PR positive vs negative	0.277	0.061	1.253	0.096
	HER2 positive vs negative	1.928	0.640	5.807	0.243
	treatment<br>FAC/FEC vs TFAC/TFEC	3.121	1.182	8.242	**0.022**
	**MNAI** Intermediate vs low	2.558	0.622	10.514	0.193
	**MNAI** High vs low	3.283	0.806	13.381	0.097
GSE22226	Age	0.951	0.884	1.024	0.186
	Tumor Size	0.967	0.831	1.125	0.664
	ER positive vs negative	1.164	0.217	6.235	0.859

	PR positive vs negative	0.366	0.067	2.001	0.246
	HER2 positive vs negative	3.855	0.877	16.948	0.074
	PAM50 basal like vs normal like	0.080	0.007	0.932	0.044
	PAM50 HER2 vs normal like	0.198	0.019	2.034	0.173
	PAM50 luminal vs normal like	0.050	0.005	0.545	0.014
	**MNAI** Intermediate vs low	1.809	0.352	9.311	0.478
	**MNAI** High vs low	6.497	1.047	40.313	**0.045**
GSE23988	ER positive vs negative	0.826	0.175	3.886	0.808
	age	0.961	0.891	1.037	0.307
	size	0.892	0.708	1.123	0.331
	LN 1 vs 0	0.544	0.126	2.343	0.414
	LN 2 vs 0	0.253	0.018	3.479	0.304
	LN 3 vs 0	628092161.830	0.000		0.999
	**MNAI** Intermediate vs low	11.942	1.123	126.968	**0.040**
	**MNAI** High vs low	30.625	2.487	377.152	**0.008**
GSE25066	age	1.012	0.989	1.036	0.308
	ER positive vs negative	1.771	0.837	3.748	0.135
	PR negative vs positive	1.010	0.513	1.989	0.977
	HER2 negative vs positive	1.469	0.230	9.396	0.685
	PAM50 basal like vs normal like	2.085	0.665	6.543	0.208
	PAM50 HER2 vs normal like	3.481	0.913	13.281	0.068
	PAM50 LumA vs normal like	7.732	2.202	27.149	**0.001**
	PAM50 LumB vs normal like	3.447	1.033	11.500	**0.044**
	**MNAI** Intermediate vs low	0.281	0.102	0.774	**0.014**
	**MNAI** High vs low	0.173	0.059	0.504	**0.001**
GSE32646	age	1.022	0.971	1.076	0.400
	ER positive vs negative	0.115	0.025	0.531	**0.006**
	PR positive vs negative	2.247	0.433	11.675	0.336
	HER2 positive vs negative	1.790	0.613	5.229	0.287
	**MNAI** Intermediate vs low	22.065	2.463	197.704	**0.006**

	**MNAI** High vs low	14.356	1.667	123.659	**0.015**
GSE41998 Ixabepilone	age	0.944	0.905	0.985	**0.008**
	ER positive vs negative	0.154	0.027	0.882	**0.036**
	PR positive vs negative	1.504	0.286	7.909	0.630
	HER2 positive vs negative	1.097	0.300	4.013	0.889
	**MNAI** Intermediate vs low	1.903	0.639	5.664	0.248
	**MNAI** High vs low	0.614	0.190	1.988	0.416
GSE41998 Paclitaxel	age	0.995	0.952	1.041	0.832
	ER positive vs negative	0.448	0.113	1.768	0.251
	PR positive vs negative	0.227	0.056	0.910	0.036
	HER2 positive vs negative	0.198	0.022	1.816	0.152
	**MNAI** Intermediate vs low	1.016	0.291	3.550	0.980
	**MNAI** High vs low	0.790	0.218	2.870	0.720
GSE42822	age	0.973	0.920	1.030	0.354
	ER positive vs negative	0.704	0.164	3.025	0.637
	PR positive vs negative	0.446	0.103	1.942	0.282
	HER2 positive vs negative	1.953	0.745	5.120	0.174
	**MNAI** Intermediate vs low	1.418	0.443	4.539	0.557
	**MNAI** High vs low	2.792	0.841	9.272	0.094
GSE50948	age	1.011	0.975	1.049	0.561
	ER positive vs negative	0.625	0.250	1.557	0.313
	PR positive vs negative	0.574	0.197	1.670	0.308
	HER2 positive vs negative	0.858	0.302	2.439	0.774
	treatment AT+CMF vs AT+CMF+T	0.285	0.120	0.674	**0.004**
	**MNAI** Intermediate vs low	2.448	0.951	6.298	0.063
	**MNAI** High vs low	3.177	1.269	7.952	**0.014**
GSE66399	Arm A vs C	0.268	0.047	1.523	0.137
	Arm B vs C	0.294	0.060	1.436	0.130
	**MNAI** Intermediate vs low	0.546	0.081	3.676	0.534
	**MNAI** High vs low	33.618	6.864	164.659	**0.000014**

GSE140494	age	0.977	0.929	1.027	0.366
	ER positive vs negative	0.582	0.074	4.557	0.606
	PR positive vs negative	0.949	0.161	5.601	0.954
	HER2 positive vs negative	0.587	0.100	3.454	0.556
	PAM50 basal like vs normal like	0.189	0.015	2.397	0.199
	PAM50 HER2 vs normal like	0.343	0.017	6.901	0.485
	PAM50 LumA vs normal like	0.093	0.010	0.859	0.036
	PAM50 LumB vs normal like	0.169	0.021	1.359	0.095
	**MNAI** Intermediate vs low	0.874	0.099	7.689	0.903
	**MNAI** High vs low	3.378	0.275	41.473	0.341
GSE163882	age	0.957	0.931	0.983	0.001
	ER positive vs negative	0.881	0.382	2.031	0.767
	PR positive vs negative	0.148	0.052	0.423	**0.00036**
	Her2 positive vs negative	3.075	1.513	6.248	**0.0019**
	**MNAI** Intermediate vs low	1.140	0.498	2.607	0.757
	**MNAI** High vs low	2.276	1.004	5.162	**0.049**
GSE173839	treatment durvalumab/olaparib vs control	0.699	0.270	1.808	0.460
	hormone receptors positive vs negative	1.237	0.484	3.160	0.657
	**MNAI** Intermediate vs low	3.308	1.070	10.221	**0.038**
	**MNAI** High vs low	8.251	2.534	26.870	**0.00046**
GSE194040 Paclitaxel	hormone receptors positive vs negative	1.035	0.435	2.462	0.938
	**MNAI** Intermediate vs low	2.815	0.837	9.463	0.094
	**MNAI** High vs low	4.411	1.282	15.184	**0.019**
GSE194040 Paclitaxel+ABT888+Carboplatin	hormone receptors positive vs negative	0.253	0.074	0.865	**0.028**
	**MNAI** Intermediate vs low	598626021.634	0.000		0.999
	**MNAI** High vs low	713462035.424	0.000		0.999
GSE194040 Paclitaxel+AMG386	hormone receptors positive vs negative	0.269	0.103	0.702	**0.007**
	**MNAI** Intermediate vs low	0.976	0.301	3.163	0.967

	**MNAI** High vs low	1.167	0.364	3.738	0.795
GSE194040<br>Paclitaxel+AMG386+Trastuzumab	hormone receptors<br>positive vs negative	0.251	0.016	3.999	0.328
	**MNAI** Intermediate vs low	1.989	0.216	18.274	0.544
	**MNAI** High vs low	0.000	0.000		0.999
GSE194040<br>Paclitaxel+Ganetespib	hormone receptors<br>positive vs negative	0.396	0.130	1.207	0.103
	**MNAI** Intermediate vs low	1.674	0.256	10.948	0.591
	**MNAI** High vs low	4.273	0.777	23.501	0.095
GSE194040<br>Paclitaxel+Ganitumab	hormone receptors<br>positive vs negative	0.584	0.204	1.673	0.317
	**MNAI** Intermediate vs low	7.719	0.895	66.608	0.063
	**MNAI** High vs low	10.887	1.203	98.530	**0.034**
GSE194040<br>Paclitaxel+MK-2206	hormone receptors<br>positive vs negative	0.294	0.083	1.046	0.059
	**MNAI** Intermediate vs low	0.902	0.206	3.959	0.892
	**MNAI** High vs low	0.756	0.170	3.367	0.713
GSE194040<br>Paclitaxel+MK-2206+Trastuzumab	hormone receptors<br>positive vs negative	0.211	0.047	0.948	**0.042**
	**MNAI** Intermediate vs low	1.599	0.312	8.196	0.574
	**MNAI** High vs low	0.986	0.099	9.838	0.991
GSE194040<br>Paclitaxel+Neratinib	hormone receptors<br>positive vs negative	0.379	0.156	0.923	**0.033**
	HER2	2.488	0.888	6.971	0.083
	**MNAI** Intermediate vs low	2.558	0.923	7.091	0.071
	**MNAI** High vs low	2.129	0.615	7.371	0.233
GSE194040<br>Paclitaxel+Pembrolizumab	hormone receptors<br>positive vs negative	0.369	0.120	1.137	0.083
	**MNAI** Intermediate vs low	1.915	0.530	6.917	0.321
	**MNAI** High vs low	4.729	1.192	18.755	**0.027**
GSE194040<br>Paclitaxel+Pertuzumab+Trastuzumab	hormone receptors<br>positive vs negative	0.242	0.051	1.157	0.076
	**MNAI** Intermediate vs low	2.913	0.678	12.507	0.150
	**MNAI** High vs low	2.247	0.183	27.618	0.527

GSE194040<br>Paclitaxel+Trastuzumab	hormone receptors<br>positive vs negative	0.188	0.029	1.236	0.082
	**MNAI** Intermediate vs low	1.725	0.254	11.699	0.577
	**MNAI** High vs low	0.000	0.000		0.999
GSE194040<br>T-DM1+Pertuzumab	hormone receptors<br>positive vs negative	0.481	0.121	1.909	0.298
	**MNAI** Intermediate vs low	2.441	0.691	8.617	0.166
	**MNAI** High vs low	0.812	0.112	5.873	0.837
